# Cigarette type or smoking history: Which has a greater impact on the metabolic syndrome and its components?

**DOI:** 10.1038/s41598-020-67524-2

**Published:** 2020-06-26

**Authors:** Sarah Soyeon Oh, Ji-Eun Jang, Doo-Woong Lee, Eun-Cheol Park, Sung-In Jang

**Affiliations:** 1 Institute of Health Services Research, Yonsei University College of Medicine, Seoul, Republic of Korea; 2 Department of Public Health, Graduate School, Yonsei University College of Medicine, Seoul, Republic of Korea; 3 Department of Preventive Medicine, Yonsei University College of Medicine, Seoul, Republic of Korea; 4 Department of Public Health, Graduate School, Ajou University College of Medicine, Seoul, Republic of Korea

**Keywords:** Health care, Risk factors

## Abstract

Few studies have researched the gender-specific effects of electronic nicotine delivery systems on the metabolic syndrome (MetS) and/or its risk factors (central obesity, raised triglycerides, decreased HDL cholesterol, raised blood pressure, raised fasting plasma glucose). Thus, this study investigated the association between smoking behavior (cigarette type, smoking history) and MetS in a nationally representative sample of Korean men and women. Our study employed data for 5,462 cases of MetS and 12,194 controls from the Korea National Health and Nutritional Examination Survey (KNHANES) for the years 2014 to 2017. Logistic regression analysis was employed to determine the association between type of cigarette (non-smoker, ex-smoker, and current smoker—conventional only, current smoker—conventional and electronic) and the prevalence of metabolic syndrome and its risk factors. Smoking history was clinically quantified by pack-year. No association between cigarette type and MetS was found for men. For women, relative to non-smokers, smokers of conventional cigarettes (OR 1.80, 95% CI 1.02–3.18) and both conventional and electronic cigarettes (OR 4.02, 95% CI 1.48–10.93) had increased odds of MetS. While there was no association between smoking history and MetS for women, for men, conventional smoking history was associated with MetS for individuals with a smoking history of > 25 pack-years (> 25 to ≤ 37.5 OR 1.45, 95% CI 1.04–2.02; > 37.5 to ≤ 50 OR 1.53, 95% CI 1.08–2.18; > 50 OR 1.56, 95% CI 1.07–2.27). Sex differences were found in the association between smoking behavior and MetS. Such findings reveal sociodemographic differences that should be considered for interventions regarding conventional and/or e-cigarette users at risk of metabolic complications.

## Introduction

The metabolic syndrome (MetS) and its risk factors (central obesity, raised triglycerides, decreased HDL cholesterol, raised blood pressure, raised fasting plasma glucose) have been vital in helping identify individuals at risk of type 2 diabetes and cardiovascular disease (CVD). Although a catalyst for heart disease, lipid problems, hypertension, dementia, cancer, polycystic ovarian syndrome, and non-alcoholic fatty liver disease^1^, the mechanisms underlying the development of MetS remain obscure. What is certain is that a growing body of literature asserts that various lifestyle factors including smoking, alcohol consumption^2^, diet, and physical activity^3^ contribute to its onset.

Electronic nicotine delivery systems, also known as e-cigarettes, are battery-powered products that deliver nicotine in the form of an aerosol^4^. Currently, the ever use of e-cigarettes is 8.5% in the United States and 6.6% in South Korea^5^. Often advertised as a “healthier” alternatives to conventional cigarettes or smoking cessation aids, e-cigarettes purportedly do not involve tobacco combustion and therefore, have reduced toxicant exposure compared to traditional cigarettes^6^.

However, research on long-term toxicity has been limited and studies attempting to show the efficacy of e-cigarettes as a healthier alternative to conventional smoking have had mixed results^6^. In particularly, while many studies have found a positive association between conventional cigarette smoking and the metabolic syndrome^7–11^, few have examined the effects of e-cigarettes, on MetS and/or its risk factors.

The existing body of literature on the effects of e-cigarettes on lipid profile is both limited and inconsistent. In one study, e-cigarettes were associated with weight loss and a decrease in some participants’ blood sugar and cholesterol levels^12^. In another study, users of e-cigarettes who completely quit regular cigarette smoking were less likely to report weight gain when compared with continuing smokers and reducers^13^. In a study comparing Wistar rats administered and not administered with e-liquid with nicotine exposure, a significant decrease in cholesterol and LDL levels were found in the e-cigarette group^14^. In a recent study comparing e-cigarette smokers to non-smokers, e-cigarette use was found to be associated with systemic oxidative stress, which is an indirect catalyst of cardiovascular risk^15^.

Conversely, in another study of mice exposed to e-cigarettes, it was shown that when exposed to equivalent doses of nicotine as conventional cigarettes, the weight reducing effects of nicotine were not found^16^. In another study of the effects of e-cigarettes on heart rate and blood pressure, it was found that e-cigarettes have no effect on blood pressure or pulse among daily users^17^ although it should be noted that Konstatinos Farsalinos has a long history of funding from e-cigarette companies.

Thus, scholars have emphasized that the metabolic effects of e-cigarettes should be further evaluated, especially with direct comparisons of the health effects of e-cigarettes with those of conventional cigarettes^18^. In the present study, we examined the association between electronic cigarette and/or conventional cigarette usage and MetS. This association was also stratified for the following risk factors of MetS: central obesity, raised triglycerides, decreased HDL cholesterol, raised blood pressure, raised fasting plasma glucose.

## Materials and methods

### Study population and data

This study was conducted using data from the Korea National Health and Nutrition Examination Survey (KNHANES). The KNHANES aims to evaluate the health and nutritional status of South Koreans and provide data for the development and evaluation of health policies and programs in Korea. The survey produces statistics regarding smoking, drinking, physical activity, and obesity for the WHO^19^ and the Organization for Economic Cooperation and Development (OECD).

The KNHANES is conducted by the Korean Ministry of Health and Welfare in conjunction with the Centers for Disease Control and Prevention (CDC). Survey data is obtained by specially trained interviewers who are not provided with any prior information about participants. Surveys are performed throughout 192 regions each year and 10,000 individuals ≥ 1 year of age are targeted.

For the purpose of our study, we examined the data of 17,656 individuals who participated in the survey between the years of 2014 and 2017. 1,738 individuals diagnosed by a medical professional with type 1 or type 2 diabetes were excluded from our study, as well as subjects with missing information about cigarette type, smoking history (pack-year), and/or the risk factors of MetS.

### Measures

#### Outcome variable

In this study, the prevalence of the metabolic syndrome and its components was selected as the outcome variable. The presence of MetS was measured using the International Diabetes Federation’s definition specific to South Asians^2^. According to the IDF’s worldwide definition, those with MetS are required to be centrally obese (measured by a waist circumference of ≥ 90 cm if male and ≥ 80 cm if female), as well as have two of the following four features: (1) an increased triglyceride level of ≥ 150 mg/dL or specific treatment; (2) a decreased high density lipoprotein cholesterol level of < 40 mg/dL in men and < 50 mg/dL in women or specific treatment; (3) raised blood pressure, indicated by a systolic blood pressure of ≥ 130 mmHg, a diastolic blood pressure of ≥ 85 mmHg, or treatment of previously diagnosed hypertension; and (4) an increased fasting plasma glucose level of ≥ 100 mg/dL or previously diagnosed type 2 diabetes. Such components, as well as all health-related components of the KNHANES, were collected via standardized physical examination by medical technicians serving as staff members for the survey^2^.

#### Cigarette type

The survey asked all subjects whether they used cigarettes or e-cigarettes currently or had ever used these products during their lifetime. Accordingly, we classified the subjects into four categories: non-smoker, ex-smoker, current smoker (conventional only), and current smoker (conventional and electronic). This classification was in accordance with that of previous studies investigating cigarette type with the same survey instrument^20^.

### Covariates

Demographic, socioeconomic, and health-related covariates were included in this study. Covariates included age (20–29, 30–39, 40–49, 50–59, 60–69, ≥ 70), smoking history (≤ 5, > 5 to ≤ 10, > 10 to ≤ 15, > 15 to ≤ 20, > 20 to ≤ 25, > 25 to ≤ 37,0.5, > 37.5 to ≤ 50, > 50)^21^, level of physical activity (low, high), region (urban, rural), high-risk drinking (no, yes), menopause (no, yes- natural, yes-sartificial)^22^, serum hs-CRP levels (low: less than or equal to 3.0 mg/L, high: above 3.0 mg/L)^23^, household income group (low, medium–low, medium–high, high), occupation (white collar, sales and services, blue collar), and educational attainment (≤ elementary school, middle school, high school diploma, ≥ bachelor’s degree) and year of survey (2014, 2015, 2016, 2017). The International Physical Activity Questionnaire was adopted to determine the level of physical activity. “High” physical activity was defined as ≥ 20 min of vigorous-intensity physical activity for ≥ 3 days a week, or ≥ 30 min of light- or moderate-intensity physical activity ≥ 5 days a week^24^. Income groups were obtained by dividing household income by the square root of the number of members in a household, which is the standard method recommended by the Organization for Economic Cooperation and Development (OECD)^25^.

### Statistical analysis

To examine the association between cigarette type and MetS, as well as its risk factors, multivariate logistic regression analysis was performed using weighted data, while controlling for all demographic, socioeconomic, and health-related covariates. Odds ratios and 95% confidence intervals^26^ were calculated to compare non-smokers with ex-smokers, current smokers (conventional only), and current smokers (conventional and electronic). The calculated p-values in this study were considered significant if lower than 0.05. All analyses were performed using SAS software, version 9.4 (SAS Institute, Cary, North Carolina, USA).

## Results

Table 1 presents the general characteristics of our study population. 5,462 cases of MetS and 12,194 controls were analyzed from the Korea National Health and Nutritional Examination Survey (KNHANES) for the years 2014 to 2017. Among cases of MetS, 45.9% were males and 54.1% were females. Among controls, 37.6% were males and 62.4% were females.

**Table 1:** General characteristics of study observations by cases of metabolic syndrome and controls (2014–2017).

	Total	Cases (n = 5,462)				p-value	Total	Controls (n = 12,194)				p-value
		Male		Female				Male		Female		
		N	%	N	%			N	%	N	%	
**Cigarette type**												
Non-smoker	3,193	499	(15.6)	2,694	(84.4)	< 0.0001	8,182	1,321	(16.1)	6,861	(83.9)	< 0.0001
Ex-smoker	1,293	1,154	(89.2)	139	(10.8)		2,420	1,941	(80.2)	479	(19.8)	
Current smoker (conventional only)	893	781	(87.5)	112	(12.5)		1,432	1,189	(83.0)	243	(17.0)	
Current smoker (conventional and electronic)	83	74	(89.2)	9	(10.8)		160	134	(83.8)	26	(16.3)	
**Smoking history (pack-years)**												
Non-smoker	3,284	563	(17.1)	2,721	(82.9)	< 0.0001	8,466	1,492	(17.6)	6,974	(82.4)	< 0.0001
≤ 5	394	280	(71.1)	114	(28.9)		1,230	773	(62.8)	457	(37.2)	
> 5 to ≤ 10	296	257	(86.8)	39	(13.2)		643	550	(85.5)	93	(14.5)	
> 10 to ≤ 15	283	257	(90.8)	26	(9.2)		467	422	(90.4)	45	(9.6)	
> 15 to ≤ 20	290	269	(92.8)	21	(7.2)		372	356	(95.7)	16	(4.3)	
> 20 to ≤ 25	192	186	(96.9)	6	(3.1)		260	251	(96.5)	9	(3.5)	
> 25 to ≤ 37.5	385	371	(96.4)	14	(3.6)		412	403	(97.8)	9	(2.2)	
> 37.5 to ≤ 50	203	197	(97.0)	6	(3.0)		212	207	(97.6)	5	(2.4)	
> 50	135	128	(94.8)	7	(5.2)		132	131	(99.2)	1	(0.8)	
**Age**												
20–29	188	134	(71.3)	54	(28.7)	< 0.0001	2,033	826	(40.6)	1,207	(59.4)	< 0.0001
30–39	572	371	(64.9)	201	(35.1)		2,605	857	(32.9)	1,748	(67.1)	
40–49	922	530	(57.5)	392	(42.5)		2,584	852	(33.0)	1,732	(67.0)	
50–59	1,302	586	(45.0)	716	(55.0)		2,345	823	(35.1)	1,522	(64.9)	
60–69	1,426	541	(37.9)	885	(62.1)		1,609	693	(43.1)	916	(56.9)	
≥ 70	1,052	346	(32.9)	706	(67.1)		1,018	534	(52.5)	484	(47.5)	
**Level of physical activity**												
Low	3,187	1,337	(42.0)	1,850	(58.0)	< 0.0001	5,995	2,111	(35.2)	3,884	(64.8)	< 0.0001
High	2,275	1,171	(51.5)	1,104	(48.5)		6,199	2,474	(39.9)	3,725	(60.1)	
**Region**												
Urban	2,086	982	(47.1)	1,104	(52.9)	0.177	5,148	1,864	(36.2)	3,284	(63.8)	0.007
Rural	3,376	1,526	(45.2)	1,850	(54.8)		7,046	2,721	(38.6)	4,325	(61.4)	
**High-risk drinking**												
No	4,667	1,853	(39.7)	2,814	(60.3)	< 0.0001	11,119	3,886	(34.9)	7,233	(65.1)	< 0.0001
Yes	795	655	(82.4)	140	(17.6)		1,075	699	(65.0)	376	(35.0)	
**Menopause (females only)**												
No	723	0	(0.0)	723	(100.0)	-	4,831	0	(0.0)	4,831	(100.0)	-
Yes: Natural	1,920	0	(0.0)	1,920	(100.0)		2,403	0	(0.0)	2,403	(100.0)	
Yes: Artificial	311	0	(0.0)	311	(100.0)		375	0	(0.0)	375	(100.0)	
**Serum hs-CRP**												
Low: less than or equal to 3.0 mg/L	4,966	2,262	(45.5)	2,704	(54.5)	0.0846	11,632	4,310	(37.1)	7,322	(62.9)	< 0.0001
High: above 3.0 mg/L	496	246	(49.6)	250	(50.4)		562	275	(48.9)	287	(51.1)	
**Household income**												
Low	1,258	412	(32.8)	846	(67.2)	< 0.0001	1,511	601	(39.8)	910	(60.2)	0.2392
Medium–low	1,452	620	(42.7)	832	(57.3)		2,837	1,077	(38.0)	1,760	(62.0)	
Medium–high	1,402	690	(49.2)	712	(50.8)		3,742	1,386	(37.0)	2,356	(63.0)	
High	1,350	786	(58.2)	564	(41.8)		4,104	1,521	(37.1)	2,583	(62.9)	
**Occupation**												
White collar	1,715	994	(58.0)	721	(42.0)	< 0.0001	5,175	1,959	(37.9)	3,216	(62.1)	< 0.0001
Sales and services	1,551	890	(57.4)	661	(42.6)		2,521	1,518	(60.2)	1,003	(39.8)	
Blue collar	2,196	624	(28.4)	1,572	(71.6)		4,498	1,108	(24.6)	3,390	(75.4)	
**Educational attainment**												
≤ Elementary school	1,694	398	(23.5)	1,296	(76.5)	< 0.0001	1,552	531	(34.2)	1,021	(65.8)	0.0065
Middle school	717	293	(40.9)	424	(59.1)		1,086	440	(40.5)	646	(59.5)	
High school diploma	1,578	798	(50.6)	780	(49.4)		4,206	1,608	(38.2)	2,598	(61.8)	
≥ Bachelor's degree	1,473	1,019	(69.2)	454	(30.8)		5,350	2,006	(37.5)	3,344	(62.5)	
**Year**												
2014	846	272	(32.2)	574	(67.8)	< 0.0001	2,610	759	(29.1)	1,851	(70.9)	< 0.0001
2015	1,260	559	(44.4)	701	(55.6)		3,035	1,251	(41.2)	1,784	(58.8)	
2016	1,386	651	(47.0)	735	(53.0)		3,553	1,441	(40.6)	2,112	(59.4)	
2017	1,970	1,026	(52.1)	944	(47.9)		2,996	1,134	(37.9)	1,862	(62.1)	
**Total**	5,462	2,508	(45.9)	2,954	(54.1)		12,194	4,585	(37.6)	7,609	(62.4)	

Table 2 presents the factors associated with MetS, found through the results of our logistic regression analysis. While there was no association between cigarette type and MetS for men, for women, relative to non-smokers, smokers of conventional cigarettes (OR 1.80, 95% CI 1.02–3.18) and both conventional and electronic cigarettes (OR 4.02, 95% CI 1.48–10.93) had increased odds of MetS. While there was no association between smoking history and MetS for women, for men, conventional smoking history was associated with MetS for individuals with a smoking history of > 25 pack-years (> 25 to ≤ 37.5 OR 1.45, 95% CI 1.04–2.02; > 37.5 to ≤ 50 OR 1.53, 95% CI 1.08–2.18; > 50 OR 1.56, 95% CI 1.07–2.27). A high level of physical activity was associated with decreased odds of MetS for both males (OR 0.88, 95% CI 0.80–0.98) and females (OR 0.85, 95% CI 0.78–0.94) compared to low, while high-risk drinking was associated with increased odds of MetS for both males (OR 1.89, 95% CI 1.67–2.14) and females (OR 1.52, 95% CI 1.21–1.91). Females with artificial menopause also had increased odds of MetS (OR 1.54, 95% CI 1.54–1.23–1.92). A serum hs-CRP level greater than 3.0 mg/L was associated with increased odds of MetS for both males (OR 1.51, 95% CI 1.26–1.80) and females (OR 2.45, 95% CI 2.01–2.98).

**Table 2:** Factors associated with the metabolic syndrome (2014–2017).*

	Male			Female		
	Odds ratio	95% CI	p-value	Odds ratio	95% CI	p-value
**Cigarette type**						
Non-smoker	1.00	–		1.00	–	
Ex-smoker	1.02	(0.75–1.38)	0.903	1.54	(0.98–2.41)	0.062
Current smoker (conventional only)	1.05	(0.75–1.46)	0.790	1.80**	**(1.02–3.18)**	**0.042**
Current smoker (conventional and electronic)	1.18	(0.76–1.82)	0.464	**4.02**	**(1.48–10.93)**	**0.006**
**Smoking history (pack-years)**						
Non-smoker	1.00	–		1.00	–	
≤ 5	0.93	(0.68**–**1.28)	0.656	0.65	(0.39**–**1.07)	0.092
> 5 to ≤ 10	0.99	(0.72**–**1.37)	0.959	0.75	(0.40**–**1.41)	0.366
> 10 to ≤ 15	1.13	(0.81**–**1.57)	0.470	0.66	(0.32**–**1.36)	0.264
> 15 to ≤ 20	1.38	(0.99**–**1.93)	0.056	1.12	(0.49**–**2.55)	0.785
> 20 to ≤ 25	1.20	(0.85**–**1.71)	0.302	0.91	(0.31**–**2.67)	0.857
> 25 to ≤ 37.5	**1.45**	**(1.04–2.02)**	**0.028**	1.03	(0.39–2.74)	0.948
> 37.5 to ≤ 50	**1.53**	**(1.08–2.18)**	**0.017**	0.90	(0.27–3.05)	0.869
> 50	**1.56**	**(1.07–2.27)**	**0.020**	2.69	(0.32–23.01)	0.366
**Age**						
20–29	1.00	–		1.00	–	
30–39	**2.62**	**(2.06–3.32)**	< 0.**0001**	**2.65**	**(1.95–3.60)**	< 0.**0001**
40–49	**3.72**	**(2.95–4.69)**	< 0.**0001**	**5.19**	**(3.87–6.95)**	< 0.**0001**
50–59	**4.28**	**(3.40–5.40)**	< 0.**0001**	**8.06**	**(5.79–11.21)**	< 0.**0001**
60–69	**4.99**	**(3.94–6.33)**	< 0.**0001**	**12.11**	**(8.51–17.22)**	< 0.**0001**
≥ 70	**4.64**	**(3.60–5.96)**	< 0.**0001**	**16.67**	**(11.57–24.04)**	< 0.**0001**
**Level of physical activity**						
Low	1.00	–		1.00	–	
High	**0.88**	**(0.80–0.98)**	**0.014**	**0.85**	**(0.78–0.94)**	**0.001**
**Region**						
Urban	1.00	–		1.00	–	
Rural	1.01	(0.92–1.12)	0.821	**1.14**	**(1.03–1.25)**	**0.008**
**High-risk drinking**						
No	1.00	–		1.00	–	
Yes	**1.89**	**(1.67–2.14)**	< 0.**0001**	**1.52**	**(1.21–1.91)**	**0.000**
**Menopause (females only)**						
No	-			1.00	–	
Yes: Natural	**-**			1.13	(0.93–1.37)	0.237
Yes: Artificial	**-**			**1.54**	**(1.23–1.92)**	**0.000**
**Serum hs-CRP**						
Low: less than or equal to 3.0 mg/L	1.00	–		1.00	–	
High: above 3.0 mg/L	**1.51**	**(1.26–1.80)**	< 0.**0001**	**2.45**	**(2.01–2.98)**	< 0.**0001**
**Household income**						
Low	1.00	–		1.00	–	
Medium–low	0.94	(0.80**–**1.10)	0.445	0.94	(0.82**–**1.08)	0.372
Medium–high	0.87	(0.73**–**1.02)	0.086	**0.83**	**(0.72–0.96)**	**0.011**
High	0.84	(0.71**–**1.00)	0.053	**0.69**	**(0.59–0.80)**	< 0.**0001**
**Occupation**						
White Collar	1.00	–		1.00	–	
Sales and Services	0.89	(0.78**–**1.01)	0.077	1.01	(0.88**–**1.16)	0.902
Blue Collar	1.08	(0.93–1.25)	0.336	**1.15**	**(1.03–1.29)**	**0.015**
**Educational attainment**						
≤ Elementary school	1.00	–		1.00	–	
Middle school	0.89	(0.74–1.08)	0.240	**0.73**	**(0.62–0.84)**	< 0.**0001**
High school diploma	1.00	(0.85–1.18)	0.973	**0.57**	**(0.49–0.66)**	< 0.**0001**
≥ Bachelor's degree	0.96	(0.80–1.15)	0.628	**0.37**	**(0.31–0.43)**	< 0.**0001**
**Year**						
2014	1.00	–		1.00	–	
2015	0.87	(0.74–1.03)	0.106	**0.84**	**(0.73–0.96)**	**0.012**
2016	1.03	(0.90–1.17)	0.688	1.02	(0.90–1.15)	0.805
2017	**2.22**	**(1.95–2.52)**	< 0.**0001**	**1.51**	**(1.33–1.72)**	< 0.**0001**

*Pseudo R-Square = males: 0.1256, females: 0.1133.**Statistically significant values have been printed in bold.

**Figure 1:**
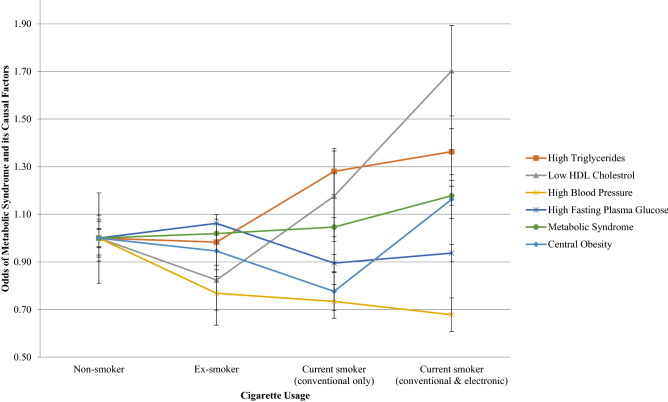
Odds of MetS and its components by cigarette usage for males.

**Figure 2:**
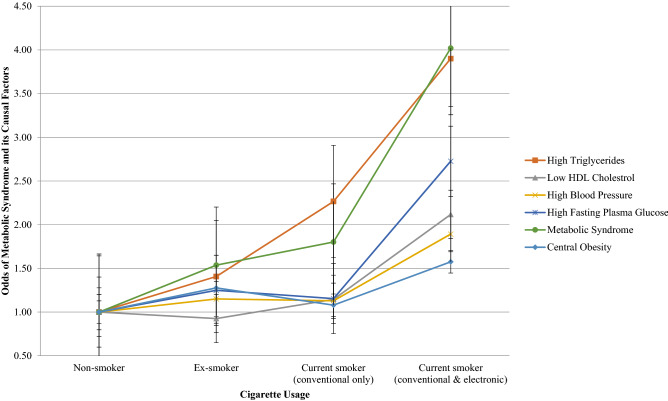
Odds of MetS and its components by cigarette usage for females.

Figures 1 and 2 show the association between cigarette usage and the odds of MetS and its components for both males and females. For females, the combined usage of both conventional and electronic cigarettes was associated with increased odds of high triglycerides (OR 3.90, 95% CI 1.54–9.89) and high fasting plasma glucose (OR 2.73, 95% CI 1.02–7.31).

## Discussion

We found that the prevalence of MetS was significantly associated with cigarette type for females only. We also found that for females, cigarette type was associated with some of the components of MetS (high triglycerides, high fasting plasma glucose levels). This was in alignment with some previous studies which show that type of both conventional and electronic cigarettes is higher in toxicity than single type of the conventional cigarette only^27,28^. Interestingly, raised blood pressure was not associated with electronic cigarette type although previous studies have noted that electronic cigarette smoking increases aortic stiffness and blood pressure in young smokers (age: 30 ± 8 years)^29^.

What is undeniable is that cigarette smoking is associated with increased total cholesterol, low-density lipoprotein, while decreasing the cardio-protective high-density lipoprotein^18^. Smoking cessation has also been associated with decreased odds of MetS after a certain period of time^18^. While our study shows no clear association between MetS and cigarette type with regard to the type of smoke used (conventional vs. conventional and electronic), the conventional smoking history variable continuously shows that a pack year of 20 or above is consistently associated with increased odds of MetS, central obesity, high triglycerides, low HDL cholesterol, high blood pressure, and high fasting plasma glucose among males. Our findings show that while cigarette type may be relevant for female users, for male users, the number and duration of cigarettes smoked may be more important.

Regarding sexual differences, previous studies have acknowledged that smoking is more strongly associated with insulin resistance and increased cardiovascular risk among women than men^28^. More research is necessary to determine how cigarette type and/or type may be more hazardous, than smoking history for women, as opposed to men. This study has several limitations that must be taken into consideration. Firstly, cigarette type, as well as variables related to smoking history and health-related behaviors were measured and classified based on self-reports, meaning that there may be various recall and information biases. A more accurate analysis would be possible if health-related behaviors could be measured through medical tests e.g. a urinary cotinine test for tobacco use. Likewise, in the KNHANES, individuals were not asked about e-cigarette use in the past, and so exclusive ex-smokers of e-cigarettes could not be separated from conventional ex-smokers in our main analysis, nor could the history or duration of e-cigarette smoking be adjusted for in pack-years.

Our study population was limited to adults, however, many studies have highlighted the popularity of e-cigarette and dual e-cigarette and tobacco use among middle and high school adolescents which future studies should take into account^28, 30^. Also, complete causal inferences are impossible to determine because the study design is cross-sectional and does not allow for lifetime trajectories of smoking, smoking cessation, and/or the development of MetS over time. Studies have shown that the risk of metabolic syndrome can persist up to 20 years after the cessation of smoking^31^. Thus, it is impossible to know if the use of e-cigarettes, and/or conventional cigarettes is responsible for the onset of MetS or its risk factors.

Likewise, some valuable but unknown or immeasurable confounders may have been excluded from our analysis because of the study design. Factors such as lifetime smoking trajectories, and/or the use of medications like statin were immeasurable from our data but likely had an effect on the association between cigarette type and MetS. Furthermore, because of the cross-sectional nature of our data, reverse causality bias is a concern; individuals with unhealthy profiles for MetS components may have been induced to stop smoking or not use e-cigarettes. This must be taken into account when interpreting our results. Lastly, other parameters that have recently been linked to MetS including levels of plasminogen activator inhibitor-1 (PAI-1) should be considered in future studies.

Despite these limitations, this investigation shows a statistically significant association between cigarette type and MetS, which adheres to the existing body of literature that debates over this relationship. It also implies that smoking both conventional cigarettes and electronic cigarettes is more hazardous for MetS and its causal factors, relative to not smoking, previously smoking, and /or smoking conventional cigarettes only.

Although South Korea has a relatively low prevalence rate of MetS compared to the rest of the OECD, our results suggest that the rate can be lowered even further if individuals are educated of such ideas. Such findings also reveal opportunities for intervention with regard to conventional and/or e-cigarette users at risk of metabolic complications.
